# Medial Cranial Fossa Meningioma Diagnosed as Mixed Anxiety Disorder with Dissociative Symptoms and Vertigo

**DOI:** 10.1155/2016/3827547

**Published:** 2016-08-29

**Authors:** Emin Mehmet Ceylan, Bariş Önen Ünsalver, Alper Evrensel

**Affiliations:** ^1^Department of Philosophy, Faculty of Humanities and Social Sciences, Üsküdar University, Istanbul, Turkey; ^2^Department of Psychology, Faculty of Humanities and Social Sciences, Üsküdar University, Istanbul, Turkey

## Abstract

Meningiomas are mostly benign tumors of the meninges that may stay clinically silent or present first with psychiatric symptoms only. We present a case of medial cranial fossa meningioma that was first diagnosed as mixed anxiety disorder with dissociative symptoms and vertigo. In light of the intact neurological and vestibular system examination, our patient's vertigo and depersonalization were firstly addressed as psychosomatic symptoms of the psychiatric syndrome. Despite decreased anxiety and improved mood, dissociative symptoms and vertigo were resistant to treatment which prompted further research yielding a left hemisphere localized meningioma. Resection of meningioma resulted in full remission of the patient proving it to be responsible for the etiology of the psychiatric syndrome and vertigo. We suggest that brain imaging should be performed for patients with late-onset (>50 years) psychiatric symptoms and those with treatment resistance. It is important to keep in mind always that medically unexplained symptoms may become explicable with detailed assessment and regular follow-up of the patient.

## 1. Introduction

Meningiomas are tumors arising from the meninges that are mostly benign in nature. While they may stay clinically silent, they may as well represent firstly with psychiatric symptoms [1]. It is not common practice to be suspicious about a brain tumor in any patient with psychiatric symptoms; however it must be noted that one-fifth of meningiomas occurring in the fifth decade of life may be manifested only with psychiatric symptoms [2]. If these patients whose symptoms may come to a halt with neurosurgery are not recognized by the psychiatrist, then they will face prolonged suffering which may bring about clinical depression due to helplessness [3]. What is more, these patients may be stigmatized as “difficult cases” by their exhausted clinicians, which may harm the therapeutic bond contributing to the further helplessness of the patient.

We present a case of meningioma that was discovered only after cranial MRI was performed for treatment resistant vertigo and dissociative symptoms.

## 2. Case Presentation

55-year-old single female patient had symptoms of depressed mood, reluctance, anxiety, difficulties in social relations and work, feelings of nearly fainting, vertigo provoked by sudden movements of the head, and hypertension. She had these symptoms for the past few years, and she had described herself as “someone who does not know her place in this world” and “someone who cannot walk firmly on solid ground.” She was experiencing some brief episodes of disorientation during which she was feeling unable to grasp the time, place, and herself as a whole in that particular moment. She had said “I do not know whether I'm floating in the sky or walking on the ground.” She had never been married and was responsible for taking care of her mother and aunt. She had features of obsessive-compulsive personality. Her vestibular system and general neurological examinations were found to be intact and therefore she was referred to psychiatry. She was diagnosed as mixed anxiety-depressive disorder. Her hypertension was managed via restricted salt intake and daily walks of at least 1-hour duration. She was started on sertraline 50 mg/day and her symptoms decreased by 40% in 2 weeks; however her vertigo and dizziness continued even at 3 months. Electroencephalography (EEG) was in the normal range and therefore cranial magnetic resonance imaging (MRI) was performed. MRI revealed a 3 × 3 × 2 cm mass of probable meningioma that was situated in left medial cranial fossa, anterior to temporal lobe, attached widely to the sphenoidal channel, neighbouring left cavernous sinus, which was markedly compressing left medial temporal area, hypothalamus, and amygdala. Left temporal lobe white matter looked edematous due to this compression. She was operated on successfully without any neurological sequela and she was started on levetiracetam for prevention of operation induced seizures. Her symptoms of vertigo and dizziness remitted and she described herself as “a person who knows her place in this life, who can walk firmly on the ground.”

Sertraline was tapered off completely and she is on levetiracetam 500 mg/day still in full remission.

## 3. Discussion

We presented a case of meningioma that was first diagnosed as mixed anxiety-depressive disorder with benign positional vertigo that were explained in the context of strenuous life conditions and personality features. In light of the intact neurological and vestibular system examination, our patient's vertigo and depersonalization were firstly addressed as psychosomatic symptoms of the psychiatric syndrome.

Despite decreased anxiety and improved mood, dissociative symptoms and vertigo were resistant to treatment which prompted further research yielding a left hemisphere localized meningioma. Resection of meningioma resulted in full remission of the patient proving it to be responsible for the etiology of the psychiatric syndrome and vertigo.

Previously reported meningioma cases that present solely with psychiatric symptoms are mostly located in the frontal lobes [1]; however our patient's meningioma was situated in left medial cranial fossa. The localization of the meningioma and further compression of limbic structures and associated peritumoral edema might be responsible for patient's symptoms. Available literature supports the association of peritumoral edema in meningioma patients and psychiatric symptoms [4] and dissociative symptoms in temporal lobe pathologies [5]. Positional vertigo has also been reported in patients whose meningioma was localized in various brain areas including the temporal area [6]. Patient's reluctance and depressed mood can be explained in the context of prolonged and unpredictable sudden dissociative symptoms and vertigo, because experiencing these symptoms may increase the person's feelings of insecurity, enhance avoidance behaviours, and result in depressed mood and reluctance [7, 8]. In our search of literature, we found only one other case similar to our patient where generalized anxiety disorder was reported in a patient with left temporal lobe meningioma [9].

The patient was started on levetiracetam to prevent postoperative seizures. Levetiracetam has been suggested as one of the emerging drugs for the treatment of panic disorder [10]; therefore it must be noted that the patient's decreased anxiety may not just be attributed to the meningioma resection but also to levetiracetam.

This case adds to the literature of meningiomas presenting only with psychiatric symptoms. We suggest that brain imaging should be performed for patients with late-onset (>50 years) psychiatric symptoms and those with treatment resistance. It is important to keep in mind always that medically unexplained symptoms may become explicable with detailed assessment and regular follow-up of the patient. Being extra alert for organicity especially in patients with psychosomatic symptoms shall improve clinical care and quality of life of the patient.
